# Thyroid Hormones Regulate Selenoprotein Expression and Selenium Status in Mice

**DOI:** 10.1371/journal.pone.0012931

**Published:** 2010-09-22

**Authors:** Jens Mittag, Thomas Behrends, Carolin S. Hoefig, Björn Vennström, Lutz Schomburg

**Affiliations:** 1 Department of Cell and Molecular Biology, Karolinska Institutet, Stockholm, Sweden; 2 Charité Universitätsmedizin, Institut für Experimentelle Endokrinologie, Berlin, Germany; University of Valencia, Spain

## Abstract

Impaired expression of selenium-containing proteins leads to perturbed thyroid hormone (TH) levels, indicating the central importance of selenium for TH homeostasis. Moreover, critically ill patients with declining serum selenium develop a syndrome of low circulating TH and a central downregulation of the hypothalamus-pituitary-thyroid axis. This prompted us to test the reciprocal effect, i.e., if TH status would also regulate selenoprotein expression and selenium levels. To investigate the TH dependency of selenium metabolism, we analyzed mice expressing a mutant TH receptor α1 (TRα1+m) that confers a receptor-mediated hypothyroidism. Serum selenium was reduced in these animals, which was a direct consequence of the mutant TRα1 and not related to their metabolic alterations. Accordingly, hyperthyroidism, genetically caused by the inactivation of TRβ or by oral TH treatment of adult mice, increased serum selenium levels in TRα1+m and controls, thus demonstrating a novel and specific role for TRα1 in selenium metabolism. Furthermore, TH affected the mRNA levels for several enzymes involved in selenoprotein biosynthesis as well as serum selenoprotein P concentrations and the expression of other antioxidative selenoproteins. Taken together, our results show that TH positively affects the serum selenium status and regulates the expression of several selenoproteins. This demonstrates that selenium and TH metabolism are interconnected through a feed-forward regulation, which can in part explain the rapid parallel downregulation of both systems in critical illness.

## Introduction

Thyroid hormones (THs) are important regulators of development and metabolism [1], [2]. Their action is mainly exerted by two nuclear TH receptors (TRs), TRα and TRβ, which are potent regulators of gene transcription in both the presence and absence of ligands [3]. Circulating TH levels are tightly controlled by the feedback regulatory system of the hypothalamus-pituitary-thyroid (HPT) axis [4]. Yet, a number of environmental agents have been described capable of interfering with HPT axis regulation [5]. In addition, extreme physiological situations have been shown to affect the HPT axis and its set points including fasting [6], neurological diseases [7] and abnormal light-dark cycles [8]. However, one of the most intensively studied perturbations of TH feedback regulation is found in critically ill patients and has been described as “low T3 syndrome”, “nonthyroidal illness” or “euthyroid-sick syndrome” [9], [10]. Here, a central downregulation of the HPT-axis takes place which is characterized by low TSH and reduced triiodothyronine (T3) and thyroxine (T4) levels [11]. Interestingly, circulating selenium (Se) concentrations decline in parallel to the deranged HPT-axis in critical illness [12]. As Se is required for the 21^st^ proteinogenic amino acid selenocysteine, it has been proposed that the impaired expression of selenoproteins such as the T4 activating 5′-deiodinase (Dio) type 1 and 2 isozymes in combination with an induced activity of the TH-inactivating 5-Dio type 3 underlie the altered serum TH pattern [11]. Especially in the hypothalamus, a disturbed expression of Se-dependent Dio might contribute to the aetiology of the syndrome [13], but the exact molecular alterations have not been fully clarified [14].

In critically ill patients, both the low serum Se- and low T3-concentrations represent negative prognostic markers for survival [15], [16]. Unfortunately, Se supplementation trials in such patients have failed to improve TH metabolism and normalize the feedback system [17]. We thus hypothesized that the reduced TH levels are not only the consequence of the low serum levels of Se, but also contribute causally to the development of the syndrome. To investigate the role of TH and TRs in the regulation of Se metabolism, we used mice heterozygous for the mutant TRα1R384C (TRα1+m mice). The chosen mutation reduces the affinity of TRα1 to the ligand T3 10-fold, thus conferring a receptor-mediated hypothyroid state specifically for TRα1 under otherwise euthyroid conditions [18]. A particular advantage of the animal model is also that the mutant TRα1 can be reactivated by supraphysiological doses of TH, either by oral treatment or endogenously by crossbreeding to hyperthyroid TRβ mice, thus allowing the differentiation between TRα1 and TRβ actions.

Using murine model systems, we here demonstrate that TH positively regulates serum Se and selenoprotein P (Sepp) levels. Our results imply a self-amplifying cycle of decreasing TH levels causing reduced Se availability, which in turn impairs the activation of T4 by the Se-dependent Dios and the Se dependent function of the HPT axis.

## Methods

### Ethics Statement

Animal care procedures were in accordance with the guidelines set by the European Community Council Directives (86/609/EEC). Required permissions were obtained from the local ethical committee (Stockholms Norra Djurförsöksetiska Nämnd, No 74/07).

### Experimental Animals

The mouse strain carrying the dominant-negative R384C mutation in TRα1 has been described previously [18]. The TRα1+m mice used for the experiments have been backcrossed to C57BL6/6NCrl for 8–10 generations. In addition, TRα1+m have been crossed to TRβ-deficient mice yielding TRα1+m TRβ−/− double mutants as described in detail previously [19]. If not indicated otherwise, littermate male mutant and wild-type mice were born by wild-type females, and 5 animals per group were used for the experiments at the age of 4–7 months. For certain experiments, wild-type and TRα1+m mice were exposed to high levels of maternal TH during embryonal development using hyperthyroid TRβ−/− mice as dams (19). The animals were housed at 21C on a 12 h light/12 h dark cycle. For thermoneutrality studies, mice were transferred to 30C at the age of 2 months and kept at this temperature for 6 weeks. If required, mice were treated with T3 via their drinking water containing 0.01% albumin and 0.5 mg/ml T3 for 12 days. Urine was collected by putting the animals on the surface of a mirror.

### Trace Element Analysis

Serum samples were diluted with ultrapure H_2_O and a Gallium standard solution was added as internal control. Tissue samples were digested in 0.1 M nitric acid for 3 hours at 150C and supplemented with the Gallium standard. A benchtop total reflection X-ray fluorescence (TXRF) photometer (Picofox™ S2, Bruker, Karlsruhe, Germany) was used to determine Se concentrations. Samples were applied to glass carriers and measured as described [20]. Intra assay CV was below 10% for a human serum reference sample (Sero, Billingstad, Norway), which was used to control quality of the measurements. Se analyzes were done in a blinded fashion with respect to the genotype and T3 treatment of the mice in a remote lab abroad from the animal facility.

### Realtime PCR

RNA was isolated from snap-frozen tissues using the RNeasy Mini Kit (Qiagen, Solna, Sweden) according to the manufacturer's instructions. Subsequent cDNA synthesis of 4 µg of RNA was carried out using Oligo(dT) Primers and the Transcriptor First Strand cDNA synthesis Kit (Roche, Stockholm, Sweden). Quantitative Realtime PCR was performed with the 7300 Real Time PCR System (Applied Biosystems, Stockholm, Sweden) and the FastStart Universal SYBR Green PCR Master Mix (Roche, Stockholm, Sweden). Specificity of amplification was verified by melting curve analyzes. A standard curve was used to correct for PCR efficiency and the results were normalized using HPRT as reference gene. The sequences of the primers used to amplify selenoprotein and Se-related enzyme transcripts are described elsewhere [21]; additional details are available on request.

### Glutathion Peroxidase (Gpx) Activity Assay

Serum, hepatic and renal Gpx activities were determined at 30C by a NADPH-coupled enzymatic test using tert-BuOOH as substrate [22] with modifications as described previously [23]. The activity was normalized against the volume (serum) or protein content (tissue) of the sample, which was determined using a commercial protein assay dye (Biorad, Sundbyberg, Sweden).

### Western Blot Analysis

Sepp concentrations in murine serum were determined by Western blot as described recently [24]. Briefly, murine serum (0.2 µl/lane) was separated in a SDS/10% polyacrylamide gel. After electrotransfer, the nitrocellulose membrane (Protran, Schleicher & Schuell, Dassel, Germany) was stained with Ponceau S (PonS) in order to control complete transfer and equal loading of the lanes. The membrane was photographed and blocked with 5% skim milk for 1 h at room temperature. Antibodies against mouse Sepp have been described previously [25] and were used at 1∶400 dilution. Secondary goat anti rabbit antibodies (DAKO, Hamburg, Germany) have been used at 1∶2000 dilution. Quantification of the PonS staining and the Western Blot were done by ImageJ, and the results show the ratio (Western Blot/PonS) normalized to the wild-type results.

### Statistics

Values are presented as means ± standard error of the mean (SEM). Statistical significance was calculated by a two-way-ANOVA followed by a Bonferroni post hoc test and considered significant if P<0.05 (*), P<0.01 (**), or P<0.001 (***).

## Results

**Figure 1:**
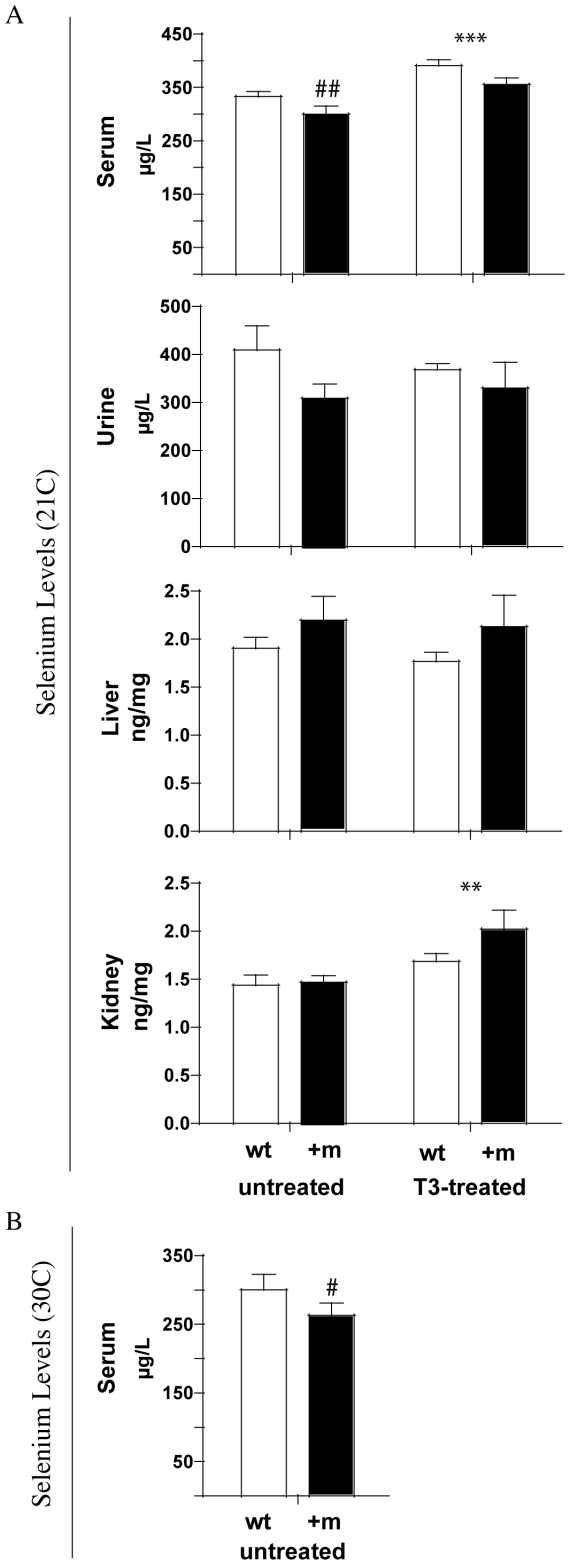
Selenium levels in wild-type and TRα1+m mutant mice with or without T3 treatment. A) Analysis of selenium concentration in serum, urine, liver and kidney of wild-type mice (wt, white bars) and animals heterozygous for a mutant TRα1 (+m, black bars), untreated or treated with supraphysiological doses of thyroid hormone (T3-treated). (##: p<0.01 for genotype, **: p<0.01 for T3 treatment, ***: p<0.001 for T3 treatment, 2-way ANOVA, n = 10 for serum, n = 5 for tissues and urine per group). B) Serum levels of selenium in wild-type (wt, white bars) and TRα1+m mice (+m, black bars) at thermoneutrality (30C). (#: p<0.05 for genotype, p = 0.39 for environmental temperature).

To test whether TH signalling affects the Se status in vivo, wild-type and TRα1+m mutant mice were analyzed. Hyperthyroidism was induced in a separate group of mice by applying T3 to their drinking water for 12 days prior to analysis. Total Se was analyzed in samples from serum, urine, liver and kidneys (Fig. 1A). Circulating Se concentrations were significantly lower in TRα1+m mice than in wild-type controls (p<0.01), and T3 increased serum Se levels by 20% in both genotypes (p<0.001). Urine and liver Se concentrations were not significantly altered in TRα1+m mice and not affected by the T3 treatment. In contrast, renal Se concentrations were significantly increased by T3 treatment. In summary, these data indicate that Se concentrations are regulated in kidney and serum in a T3- and TRα1-dependent manner.

TRα1+m mice exhibit a severe hypermetabolism at room temperature which can be normalized when the animals are reared at thermoneutrality [26]. To analyze if the increased metabolism at room temperature is the cause for the reduced serum Se concentrations observed in the mutant animals, we compared them with mice housed at 30C (Fig. 1B). The difference in serum Se concentrations persisted at thermoneutrality (p<0.05 for wild-type vs TRα1+m, p = 0.39 for room temperature vs thermoneutrality), indicating that the differences in serum Se status are unrelated to the metabolic activity.

**Figure 2:**
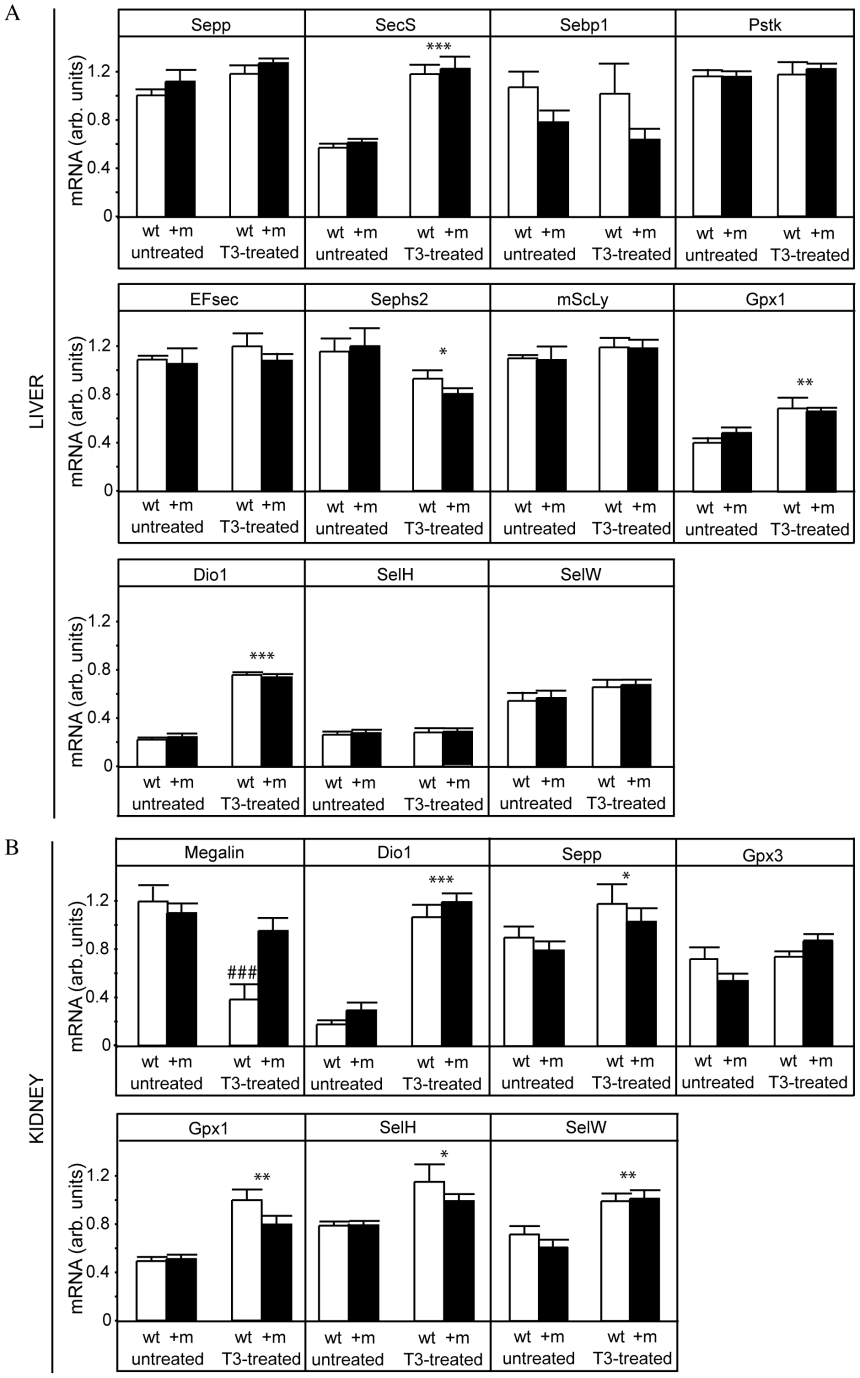
Hepatic and renal gene expression in wild-type and TRα1+m mice with or without T3 treatment. A) Expression profiling of genes involved in selenium metabolism in livers of untreated and TH treated (T3-treated) wild-type (wt, white bars) and TRα1+m mice (+m, black bars). The expression is normalized against the housekeeping gene HPRT. Sepp: selenoprotein P, SecS: selenocysteine t-RNA synthase, Sebp1: selenium binding protein 1, Pstk: phosphoseryl-tRNA kinase, EFsec: selenocysteine-specific elongation factor, Sephs2: selenophosphate-synthetase 2, mScLy: selenocysteine lyase, GPx1: glutathione peroxidase 1, Dio1: deiodinase type I, SelW/SelH: selenoprotein W or H. B) Expression profiling of genes involved in selenium metabolism in kidneys of untreated and TH treated (T3-treated) wild-type (wt, white bars) and TRα1+m mice (+m, black bars). The expression is normalized against the housekeeping gene HPRT. Dio1: deiodinases type I, Sepp: selenoprotein P, Gpx1: glutathione peroxidase 1, Gpx3: glutathione peroxidase 3, SelW/SelH: selenoprotein W or H. (###: p<0.001 for T3 treatment of the wild-type, *: p<0.05 for T3 treatment, **: p<0.01 for T3 treatment, ***: p<0.001 for T3 treatment, 2-way ANOVA with Bonferroni post hoc test, n = 5 for each group).

As serum Se concentrations are mainly controlled by hepatically-derived Sepp, the Se transport protein accounting for most of the circulating Se in both rodents and humans [27], we determined RNA levels for Sepp and other hepatic selenoproteins and enzymes involved in selenoprotein biosynthesis. We found no significant difference in any of the mRNA levels when comparing wild-type and TRα1+m mice (Fig. 2A), which is in good agreement with the lesser role of this TH receptor isoform in the liver [28]. However, a strong effect of T3 treatment was observed for Gpx1 and Dio1 mRNA in agreement with the literature [29]. Similarly, T3 also induced the expression of selenocysteine synthase SecS [30]. In contrast, transcript levels of phosphoseryl-tRNA kinase (Pstk), a dynamically regulated and limiting component of the hepatic selenoprotein biosynthesis machinery [21], [31], remained constant upon T3 stimulus, similar to selenoprotein H (SelH) and selenoprotein W (SelW) mRNA. Taken together, as hepatic Sepp, Pstk and SecS mRNA expression were not impaired, the analysis of hepatic gene expression failed to explain the decreased serum Se concentrations in TRα1+m mice and its increase upon T3 treatment.

We therefore analyzed gene expression in the kidneys as they are the major site of Se clearance. Megalin (Lrp2) is known to serve as a renal Sepp-receptor participating in Sepp binding and re-uptake from the primary filtrate in the proximal tubules [32]. Surprisingly, megalin mRNA concentrations strongly decreased in wild-type mice upon T3 treatment, but not in T3-treated TRα1+m littermates, indicating an important role for intact TRα1 signalling in the regulation of megalin mRNA expression (Fig. 2B). In parallel, mRNA levels of Sepp, Gpx1, SelH and SelW as well as Dio1 were increased in the kidney of T3-treated mutant and wild-type mice. Except for the strong effects on megalin, the T3-dependent changes of transcript levels were similar for the investigated genes in both strains of mice. It remains at present unclear whether a down-regulation of megalin transcripts by T3 contributes to altered Sepp serum levels and serum Se status in wild-type mice. As renal megalin mRNA concentrations do not follow serum Se in T3-treated TRα1+m mice, it is unlikely that this transporter alone is responsible for the different Se levels in the two genotypes.

**Figure 3:**
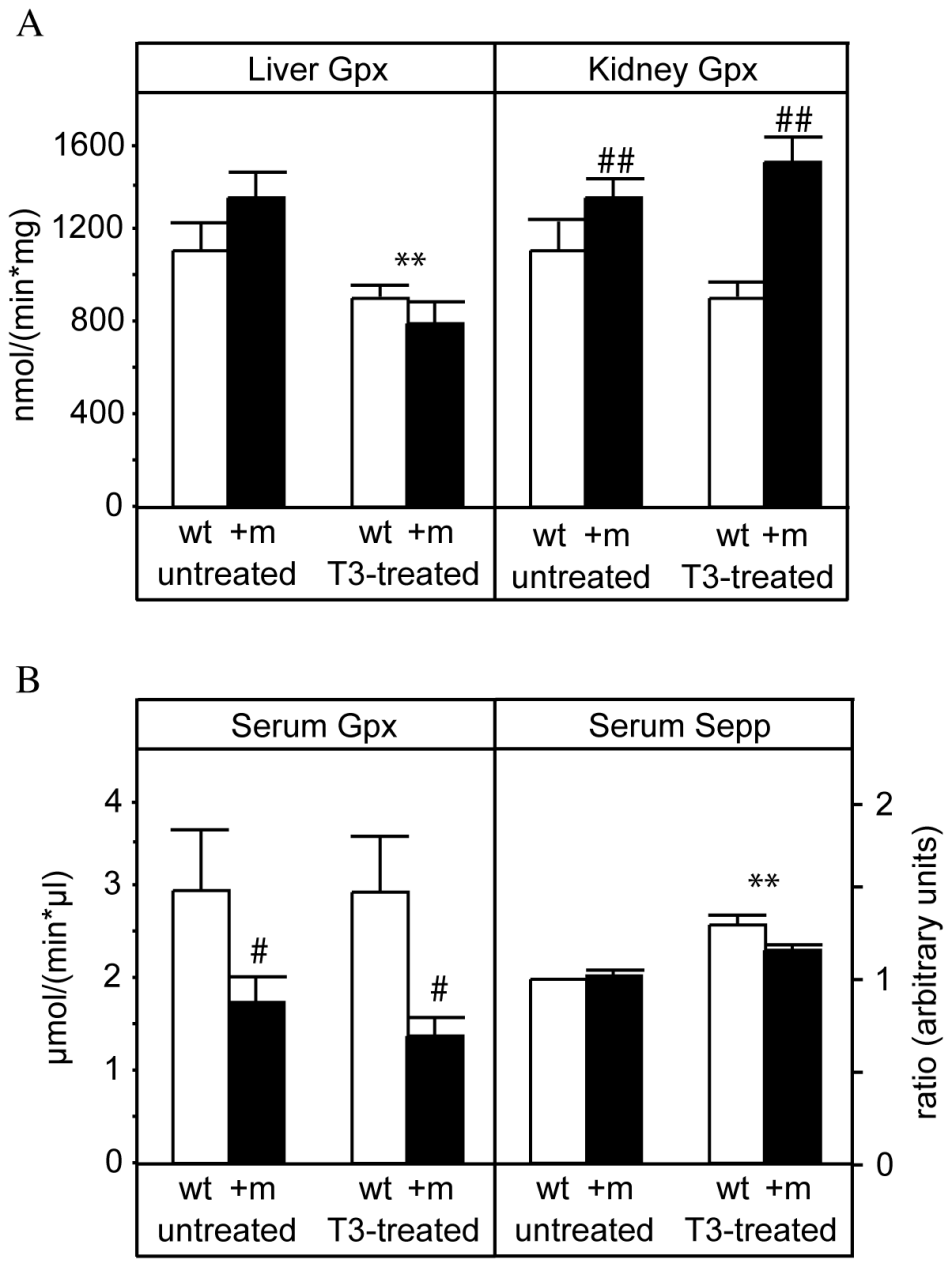
Hepatic, renal and serum glutathione-peroxidase activity and serum Sepp concentrations in wild-type and TRα1+m mice with or without T3 treatment. A) Enzymatic activity of glutathione peroxidase (Gpx) in liver and kidney of untreated and TH treated (T3-treated) wild-type (wt, white bars) and TRα1+m mice (+m, black bars). The activity is normalized against the protein content of the sample. (**: p<0.01 for T3 treatment, ##: p<0.01 for genotype, 2-way ANOVA, n = 5 for each group). B) Enzymatic activity of serum glutathione peroxidase 3 (Serum Gpx) and concentrations of selenium protein P (Serum Sepp) levels in serum of untreated and TH treated (T3-treated) wild-type (wt, white bars) and TRα1+m mice (+m, black bars). (#: p<0.05 for genotype, **: p<0.01 for T3 treatment, 2-way ANOVA).

As selenoprotein expression is strongly regulated at the post-transcriptional level, we also determined enzymatic activity of the Se-dependent Gpx as a TH target in liver and kidney (Fig. 3A). While no significant difference was observed in hepatic Gpx activity between the two genotypes, the renal activity was increased in TRα1+m mice compared to wild-type littermates. Upon T3 treatment, the activity of this enzyme decreased in the liver of both animal models, while in the kidney it was not affected by the hormone. That the effects observed on Gpx activity were not in accordance with the changes in the respective transcript levels supports previous studies demonstrating the tissue-specific regulation of selenoprotein expression at the posttranscriptional level [33].

To define the contributions of different serum Se proteins to the phenotype we analyzed the enzyme activity of Gpx3 in the serum of wild-type and TRα1+m mice with and without T3 treatment (Fig. 3B). The activity of Gpx3 was lower in TRα1+m mice, thus it is likely to contribute to the reduced serum Se in these animals. However, GPx3 activity was not altered by T3 in either genotype, indicating that this is not a direct regulation by TRα1. While no obvious difference was observed in serum Sepp between wild-type and TRα1+m mice as analyzed by Western Blot, treatment with T3 significantly increased the Sepp concentrations in the serum of both genotypes (Fig. 3B), which corresponds well with the increase observed in serum Se in these mice.

**Figure 4:**
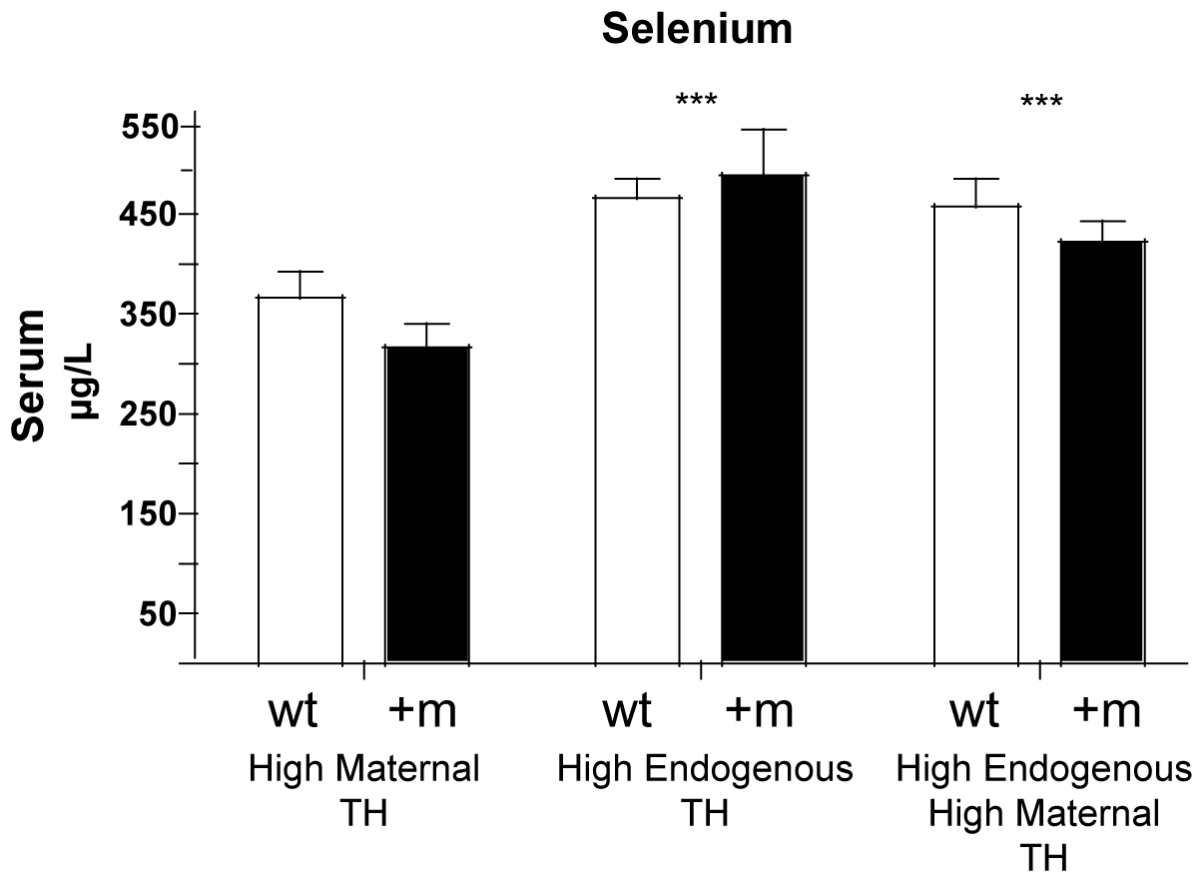
Effects of maternal or postnatal hyperthyroidism on serum selenium. Serum levels of selenium in wild-type (wt, white bars) and TRα1+m mutants (+m, black bars) born by hyperthyroid TRβ−/− mothers (high maternal TH), with inactivation of TRβ, which causes endogenous postnatal hyperthyroidism (high endogenous TH), or a combination of both (high endogenous, high maternal TH). (***: p<0.001 for T3 treatment, 2-way ANOVA, n = 5 per group).

To investigate whether the different Se levels in TRα1+m mice were a defect caused by the mutant TRα1 acting during embryonal development or a direct consequence of the TRα1R384C aporeceptor activity, we exposed the mutant mice to elevated levels of TH pre- and postnatally, thus reactivating the mutant receptor specifically in these periods (18). When the TRα1 signalling was restored during embryonal development using hyperthyroid TRβ−/− mice as dams (Fig. 4, “high maternal TH”), the TRα1+m mice still exhibited lower serum Se as adults than wild-type littermates comparable to the normal situation. However, when we reactivated the mutant TRα1 postnatally by crossing TRα1+m mice to TRβ deficient animals, which exhibit endogenously high level of TH (19), Se levels were strongly increased in hyperthyroid TRβ−/− and TRα1+m TRβ−/− double mutant animals (Fig. 4, “High Endogenous TH”). These results clearly demonstrate that Se levels are regulated by TRα1: in euthyroid TRα1+m mice, the mutant TRα1 suppresses serum Se due to its potent aporeceptor activity (comparable to the situation found in hypothyroidism), whereas high levels of TH (either by oral T3 treatment or genetically by crossbreeding to hyperthyroid TRβ−/− mice) activate TRα1 and confer an increase in serum Se even in the absence of TRβ.

## Discussion

The importance of the Se status for health and disease gains increasing recognition [34], [35], [36], [37]. Although the definition of an appropriate Se status is still debated, several biomarkers are currently in use to assess it [36], [38]. Selenoprotein enzymes have emerged as the central mediators of Se supplementation and medical Se effects. This notion is well manifested by the interaction of the family of Se-dependent Dios with TH homeostasis and metabolism studied in several animal model systems [39], [40], [41], [42]. It has been corroborated in human cross-sectional analyzes and prospective intervention studies that have addressed the effects of Se status and Se supplementation on circulating TH levels [43], [44], [45]. However, the results are not fully consistent and even in Se supplementation studies of critically ill patients with low circulating Se levels and grossly disturbed TH metabolism, a similar lack of clear effects was reported [17]. Thus, we hypothesized that serum TH levels might also affect serum Se levels, i.e. that TH and Se levels are interdependently regulated. To date the only well-established effect of TH on the selenoproteome was the regulation of the three Dio isozymes which are controlled by TH status in liver, kidney, hypothalamus and pituitary [46], [47], [48].

Our data provide a novel insight into the interactions of TH and Se metabolism. The analysis of mice heterozygous for a mutant TRα1 revealed a reduced serum Se level in these animals. Since the mutation causes a 10-fold lower affinity to the ligand T3, the receptor acts as an aporeceptor at physiological TH levels [18] and tissues expressing the mutant TRα1 exhibit a hypothyroid-like state specifically for the TRα1 isoform. Taken together with the fact that serum Se increases upon T3 treatment, our data suggest a direct regulation of serum Se by TH in a TRα1 dependent manner. This is supported by the observation that serum Se is also increased in hyperthyroid TRβ−/−mice, which excludes an involvement of TRβ.

Although the precise molecular mechanism remains yet to be elucidated, our findings provide evidence for a possible model and for the tissues involved in the regulation. First, it can be concluded that the increase in serum Se is not due to an altered Se demand of the thyroid gland, as Se levels are elevated both in T3-treated mice in which the HPT axis is suppressed and thyroid activity is reduced, and in TRβ−/− animals, which have endogenously high levels of TH due to increased thyroid activity.

Secondly, as TRα1 plays only a minor role for the hepatic mRNA expression of several selenoprotein genes such as Dio1 or GPx1, which is in line with the literature [29], [42], [49], [50], it seems unlikely that the transcription of hepatic selenoprotein genes underlies the difference in selenium metabolism between wild-type and TRα1+m mutant mice.

In contrast, the kidney seems to be an important target site of TRα1 action, since we observed a TRα1 dependent regulation of gene expression in for megalin, an important mediator of renal Se reuptake [51]. Counterintuitively, however, serum Se levels were increased in the presence of reduced megalin mRNA expression, and no increased loss of Se into the urine of T3 treated animals was observed. This suggests that megalin is not rate-limiting for efficient renal Sepp reuptake under these conditions or alternative transporters compensate for its reduction. Alternatively, an increased renal production of Se- proteins such as GPx could limit the loss of Se, which is supported by the fact that we indeed observed increased GPx activity in the kidneys of TRα1+m mutant mice. This occurs despite unaltered Gpx1 mRNA concentrations, suggesting a role for TRα1 also in the modulation of the posttranscriptional biosynthesis of selenoproteins, similar to the situation found in the sex-specific differences in hepatic selenoprotein expression [52] or the acute phase effects on liver selenoprotein biosynthesis [21].

Surprisingly, despite the increased renal GPx activity, serum GPx activity was decreased in the TRα1+m mutant mice. While this reduction most likely contributes to the difference in serum Se levels between the two genotypes, the T3 induction of serum Se seems to result from an increase of serum Sepp concentrations in both wild-type and TRα1+m mice. Since the majority of circulating Sepp is produced by hepatocytes, but hepatic Sepp mRNA levels remained unaltered upon T3 treatment, it can be speculated that TH improves the translation of Sepp in the liver. This could be achieved by an intracellular redistribution of the available Sec-tRNA away from other selenoproteins towards Sepp; a hypothesis that is supported by the fact that hepatic GPx activity declines despite increased GPx mRNA levels and unaltered or even increased total liver Se concentrations. A similar redistribution of the available Se for the posttranscriptional control of selenoprotein production has been reported during acute phase response, where decreased Sepp production and secretion is paralleled by increased selenoprotein S biosynthesis in hepatocytes despite largely unaltered transcript concentrations [24], [53]. While further analyses are required to confirm these molecular pathways, it can be concluded that TH and TRα1 affect serum Se levels by specific, yet distinct mechanisms involving hepatic and renal Sepp and GPx translation.

A limitation of the current study is however the role of TRα1 in other tissues regulating Se metabolism such as the gastrointestinal tract [54], [55]. This is caused in part by our current limitation of in-depth knowledge of this process in general and a respective lack of adequate tools and prime candidates suited to reliably reflect alterations of gastrointestinal Se uptake. Moreover, TH receptors can regulate gene expression as heterodimers with e.g. retinoic X receptor or vitamin D receptor [3], which can integrate further peripheral signals that have not been studied yet.

Nevertheless, the identified changes of serum Se, Sepp content, and GPx activity in TRα1+m mice are of clinical relevance. Although several patient families have been identified to date carrying a mutant TRβ and displaying resistance to thyroid hormone (RTH), none with a mutant TRα1 was found [56], [57]. The most likely explanation is given by the notion that the TH levels in these patients are predicted to be normal and they are thus not easily associated with a defect in TH signalling [57]. As these patients would certainly benefit from a correct diagnosis and treatment, a reliable serum parameter could facilitate their identification. Given our findings in TRα1+m mice, patients harbouring a similar mutation would exhibit decreased serum Se concentrations. As the effects have been of moderate size under normal conditions only, and mouse lines with distinct mutations in their TRα1 genes exhibit variable or sometimes even opposite phenotypic features [18], [58], [59], [60], it remains to be elucidated whether the reduced serum Se concentrations are solid screening parameters for identifying subjects with mutant TRα1 genes.

**Figure 5:**
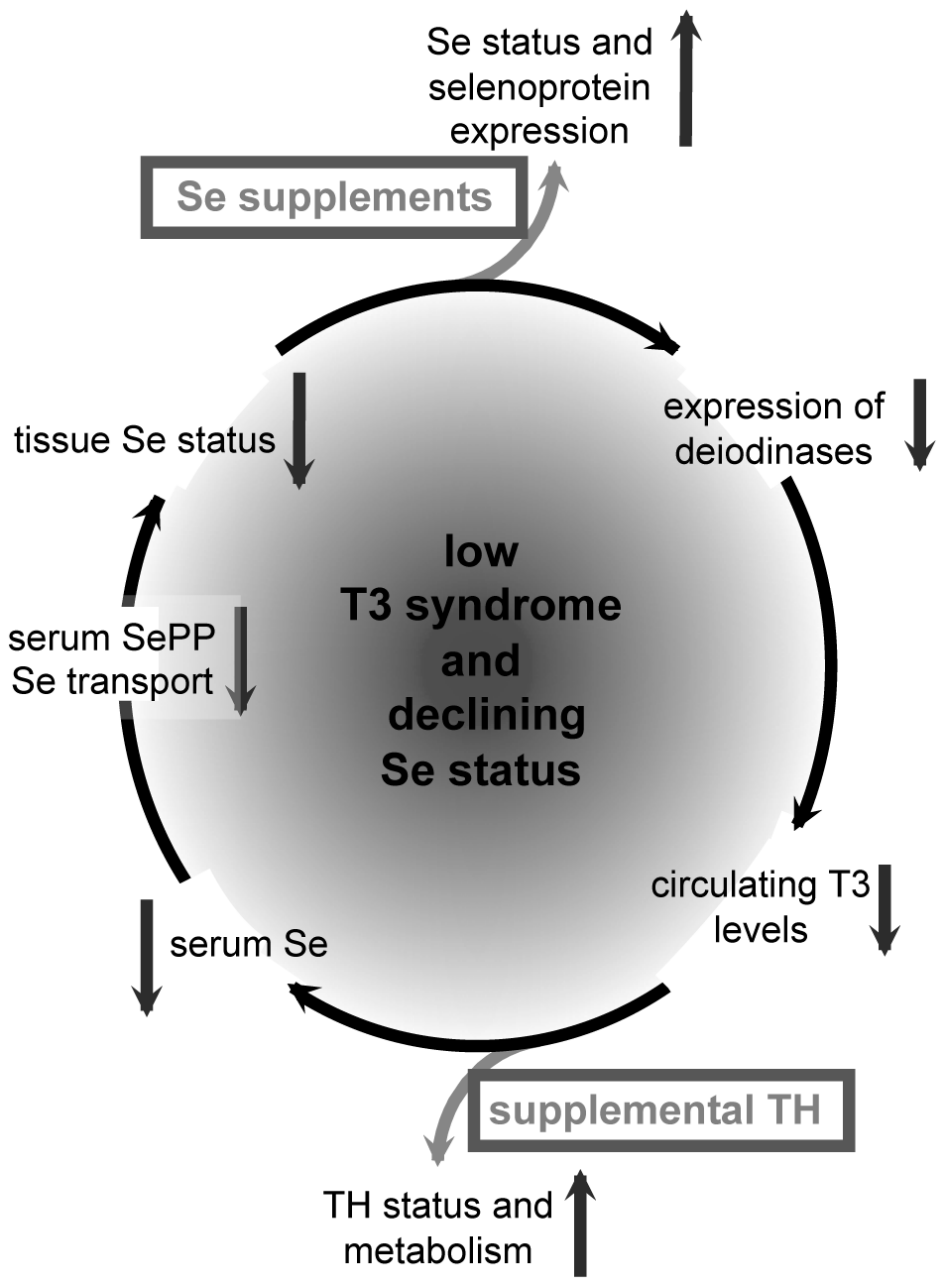
Schematic illustration of the feed-forward reaction which might take place during critical illness. Declining serum selenium concentrations cause impaired selenoprotein expression in the tissues. In parallel, impaired deiodinase expression leads to low T3 concentrations. Both pathways might aggravate each other if the declining selenium and T3 concentrations are not compensated for by a combined supplementation effort aiming to meet the patient's Se and TH requirements.

Even more relevant, our data demonstrate that serum T3 positively correlates with serum Se and that an unliganded TRα1, as in hypothyroidism, reduces serum Se levels. This interconnected feed-forward regulation of TH and Se may be of paramount pathophysiological importance in the clinics, and could contribute to the vicious cycle observed in critical illness in which both parameters are known to decline in parallel (Fig. 5). At present, rescue treatments using TH supplements have yielded controversial results in critical illness [61], [62], [63], [64]. Similarly, Se supplementation trials did not provide uniform results. Even though no trial showed adverse effects of Se, the majority of studies have been rather small and yielded either null or positive results [65]. Data from a recently completed multicentre double-blind prospective trial were explicitly positive [66], especially for the male patients enrolled [67]. Still, an intensive discussion is currently held about the best Se dosage regimen, supplement and application modus [68], [69]. Given our findings and the conflicting results for TH and Se correction trials in critically ill patients, it might be advantageous to correct T3 status and Se deficiency in parallel to interrupt the self-amplifying pathogenic mechanism aggravating the disease and all too often causing a deadly outcome (Fig. 5).
